# An Immunization Strategy for Hidden Populations

**DOI:** 10.1038/s41598-017-03379-4

**Published:** 2017-06-12

**Authors:** Saran Chen, Xin Lu

**Affiliations:** 1 College of Information System and Management, National University of Defense Technology, 410073 Changsha, P.R. China; 2 School of Mathematics and Big Data, Foshan University, 528000 Foshan, P.R. China; 3 Department of Public Health Sciences, Karolinska Institutet, 17177 Stockholm, Sweden; 4 Division of Infectious Disease, Key Laboratory of Surveillance and Early Warning on Infectious Disease, Chinese Centre for Disease Control and Prevention, Beijing, 102206 P.R. China

## Abstract

Hidden populations, such as injecting drug users (IDUs), sex workers (SWs) and men who have sex with men (MSM), are considered at high risk of contracting and transmitting infectious diseases such as AIDS, gonorrhea, syphilis etc. However, public health interventions to such groups are prohibited due to strong privacy concerns and lack of global information, which is a necessity for traditional strategies such as targeted immunization and acquaintance immunization. In this study, we introduce an innovative intervention strategy to be used in combination with a sampling approach that is widely used for hidden populations, Respondent-driven Sampling (RDS). The RDS strategy is implemented in two steps: First, RDS is used to estimate the average degree (personal network size) and degree distribution of the target population with sample data. Second, a cut-off threshold is calculated and used to screen the respondents to be immunized. Simulations on model networks and real-world networks reveal that the efficiency of the RDS strategy is close to that of the targeted strategy. As the new strategy can be implemented with the RDS sampling process, it provides a cost-efficient and feasible approach for disease intervention and control for hidden populations.

## Introduction

Hidden or hard-to-reach populations, including IDUs, SWs and MSM, are generally considered at higher risk of contracting and transmitting infectious diseases such as AIDS, gonorrhea, syphilis etc.^1–3^, Consequently, developing efficient intervention and immunization strategies for hidden populations is crucial to prevent and control the spread of these sexual transmitted diseases. However, it is difficult to access them and implement interventions due to their strong privacy and the lack of a sampling frame.

There are a number of immunization strategies developed for general populations, such as targeted strategy^4^, random strategy^5^, acquaintance strategy^5^, and other variants^6–13^. These strategies can be broadly categorized as one of two types based on whether the population information prior (e.g., the degree, degree distribution and topological structure of the whole population) is required. Global strategies can efficiently immunize the influential spreaders but require the complete knowledge of all individuals^4^. Local strategies only need local information and are less efficient than global strategies in many cases^5^. However, the hard-to-access property of hidden populations makes these traditional strategies not applicable as there is no list of a sampling frame from which influential spreaders or initial random individuals could be selected to design immunization or intervention strategies. The interventions for hidden populations typically depend on agency-based services and outreach projects^14, 15^. Although many agencies or programs provide many intervention prevention services, hidden populations often avoid using the agency-based services or are less likely to access health care due to their stigmatization and illegality^16-18^. For outreach projects, hired workers, such as current and former IDUs, are trained with professional knowledge and offer vaccinations in their communities. The basic categories are the door-to-door outreach^19, 20^, the street-based outreach^15, 21^ and the peer-driven outreach^22, 23^. It has been proven that such outreach efforts can slow down the transmission of epidemics^14, 24^, however, these methods were built from a convenience perspective and lacked in systematically targeting high influential individuals within the populations. Additionally, severe side effects of some vaccines also need a strategy for keeping the number of vaccines low.

We can see that current intervention and immunization strategy are all limited by the nature of hidden populations and lack an approach to access and target the key individuals. To overcome these limitations, in this study, we develop an efficient strategy, which is based on a sampling approach currently used widely for hidden populations, called respondent-driven sampling (RDS). RDS is a nonprobability and chain-referral sampling method, which works like snowball sampling but uses a dual incentive mechanism to stimulate the peer-driven recruitment process^25^. A typical RDS begins with a number of initial selected respondents called “seeds”. After the interview, the seeds will be given a certain number of coupons to distribute to friends and acquaintances in their social networks. Individuals with a valid coupon can participate in the process and then are given the same number of coupons to distribute. The above recruitment process is repeated until the desired sample size is reached^26^. In the recruitment process, information about who recruits whom and the respondents’ number of contacts (degree) are collected. This information can be used for correction and unbiased estimation in statistical inferences^27, 28^. RDS has been adopted widely around the world for the study of hidden populations^29, 30^, such as IDUs in India^31^, Iran^32^, SWs in China^33^, Kenya^34^, and MSM in Sweden^35^, Panama^36^. In addition, this method is approved by the World Health Organization for the surveillance of HIV^37^. The applications of RDS mainly focus on estimating the characteristics of the targeted populations and many estimators have been deveploded^38–44^.

The wide application of RDS studies provides the opportunity for us to develop a feasible intervention approach for infectious disease prevention and control in hidden populations: with the existing experimental design, it is possible to identify and immunize important individuals from the RDS sample, through which we can develop the analytical framework for the evaluation of efficiency and effectiveness of the strategy. Specifically, the basic steps of the strategy are as follows. First, we estimate the average degree (personal network size) and degree distribution of the studied population with respondents recruited through RDS. According to the estimated average degree, we obtain the approximate immunization threshold $\widehat{{g}_{c}}$ in case of targeted immunization by approximating the targeted population’s social network with a scale-free network^4^. Second, in the cumulative degree distribution *p*_*c*_(*k*) obtained from the estimated degree distribution, we can find a ‘cut-off degree’ ${k}_{cut}=\,{\rm{\max }}\,\{k|{p}_{c}(k)\le 1-\widehat{{g}_{c}}\}$. Lastly, we immunize the RDS participants whose degree *k* ≥ *k*_*cut*_. In the following, we implement simulated RDS processes on model networks and real-world social networks to achieve large enough samples for the strategy to immunize the desired number of individuals. In real applications, for which the size of sample is most likely not large enough, we discuss and compare the efficiency of RDS strategy by immunizing within the existing sample.

**Figure 1:**
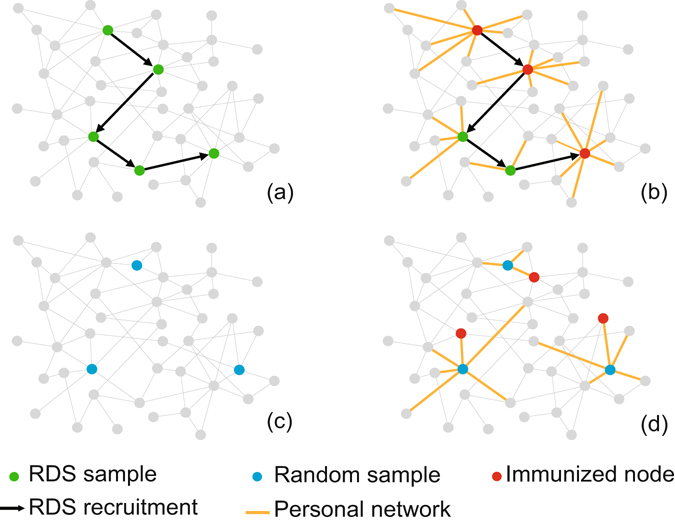
Immunization process utilising the RDS strategy with *k*_*cut*_ = 7 and a popular local immunization strategy called acquaintance immunization. In the RDS strategy, (**a**) the immunization process begins with a RDS process and (**b**) the individuals whose degree *k* ≥ 7 during the RDS chain are immunized. Compared to the RDS strategy, (**c**) the acquaintance strategy needs to randomly select individuals at the beginning and then (**d**) randomly immunizes a neighbour of the selected individual.

We consider that the RDS strategy is local because the selection of an individual to be immunized does not require the global information from the population but only depends on the comparison between the contact of the current recruitment individual and the cut-off threshold *k*_*cut*_. In contrast to traditional local strategies, the immunization process utilising the RDS strategy does not require the random selection of initial individuals as part of the first step (see Fig. 1). To verify the effectiveness of the RDS strategy, we implement the Susceptible-Infected-Susceptible (SIS) epidemiological model on model network and real-world networks.

Our study is one of the first to develop a systematically approach for hidden population immunization and intervention. Additionally, the results encourage the use of the new approach against others when there is a lack of global knowledge about the targeted population. As the new approach can be used in combination with existing RDS design, it is one of the rarely efficient and practical strategy for hidden populations.

## Results

To study the efficiency of the RDS strategy, we focused on the critical immunized fraction *f*_*c*_, which is the minimum fraction of the population required to be immunized for the eradication of epidemics, i.e., the infection’s prevalence (the fraction of infected nodes) *ρ*_*f*_ = 0. Smaller *f*_*c*_ indicates higher efficiency of a strategy. Targeted strategy, acquaintance strategy and random strategy are implemented as well for comparison.

In simulations, we look at the infection’s prevalence (the fraction of infected nodes) *ρ*_*f*_ in the stationary regime (endemic state) as a function of the fraction of immunized nodes *f*. we first immunize *f* · *N* nodes on a network of size *N* by implementing a strategy. Then we let the infection fraction for the susceptible be 0.5 (half of the susceptible nodes are infected in the network), and iterate the SIS infection process with synchronous updating^4, 5^, (see details in the last subsection of Materials and Methods). The SIS process is implemented with a fixed spreading rate *λ* = 0.25. After the system reaches the steady state, *ρ*_*f*_ in the stationary regime is obtained. Changing the value of *f*, we can obtain *ρ*_*f*_ as a function of *f*. Therefore, ${f}_{c}=\,\min \,\{\,f|{\rho }_{f}=\mathrm{0\}}$.

The configurations of the RDS strategy in simulations are as follows. We consider the basic setting for the number of seeds and coupons, i.e., 1 seed and 1 coupon because the number of seeds or coupons doesn’t change the inclusion probability of the individuals^25, 26, 45^ in RDS so that the efficiency of the RDS strategy is not affected by the seed number or coupon number (see﻿ Supplementary Fig. S3 and Fig. S4 online). Seeds are uniformly selected, and coupons are randomly distributed to the recruiter’s neighbours. RDS is implemented without replacement and the results are averaged from 100 simulation.

### Immunization in the Barabasi-Albert (BA) network

**Figure 2:**
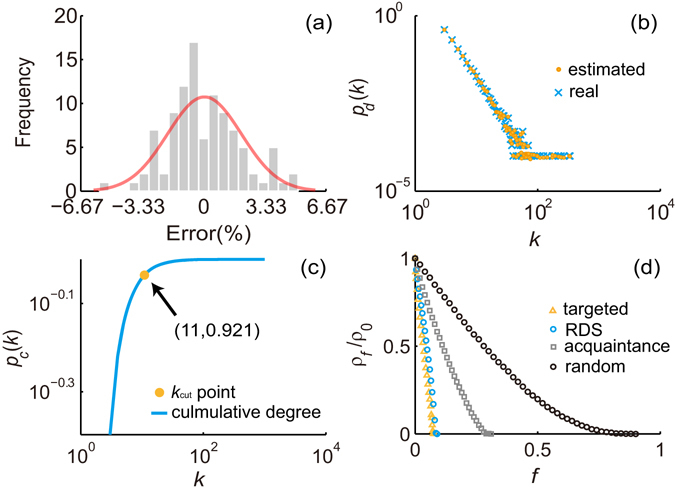
Basic results of BA network. (**a**) The error distribution of estimated average degree. (**b**) The average of estimated degree distributions and the real degree distribution. (**c**) The cumulative degree distribution obtained from the estimated degree distribution of one simulation. In this simulation, the value of *k*_*cut*_ is 11. (**d**) Reduced prevalence *ρ*_*f*_/*ρ*_0_ from simulations of the SIS model on the BA network with random strategy, acquaintance strategy, targeted strategy and RDS strategy, at a fixed spreading rate *λ* = 0.25. The prevalence is averaged over 100 simulations.

We first implemented the RDS strategy in the Barabasi-Albert (BA) network^46^. Figure 2(a) shows the error distribution of 100 estimated results of average degree. All the absolute errors were less than 6.67%; and 98% of the absolute errors were less than 5%. Figure 2(b) shows the average of 100 estimated results of degree distribution. Both results indicate that the network degree can be well approximated by estimates generated by the RDS estimator from the sample. Figure 2(c) shows the estimated cumulative degree distribution of one simulation. The corresponding estimated average degree <*k*> was 6.001. According to equation((3)), we can obtain the estimated immunization threshold $\widehat{{g}_{c}}=0.07$. In the cumulative degree distribution curve, we can find the ${k}_{cut}=\,{\rm{\max }}\,\{k|{p}_{c}(k)\le \mathrm{0.93\}}=11$. The results of numerical simulations for spread of the epidemic in the BA network are shown in Fig. 2(d). The reduced prevalence *ρ*_*f*_/*ρ*_0_ (*ρ*_0_ is the infection’s prevalence without immunization)^4^ for targeted strategy and RDS strategy display a sharp drop and their efficiencies are much better than that of acquaintance strategy and random strategy. We can see that, in the simulated BA network, the efficiency of the RDS strategy, which immunizes nodes from the sample drawn from the RDS process, is almost the same as that of the targeted strategy, which requires global information (degree of each node) for the network. Specifically, the simulation results of the critical immunized fraction *f*_*c*_ for RDS strategy and targeted strategy are very close: about 0.08 for RDS strategy and about 0.07 for targeted strategy. That is to say, to eradicate epidemics, the fraction of nodes that RDS strategy needs to immunize is about the same as that observed for the targeted strategy, which is much less than the immunization fraction required by the acquaintance strategy (*f*_*c*_ = 0.31) and the random strategy (*f*_*c*_ = 0.9).

### Immunization in real-world networks

**Figure 3:**
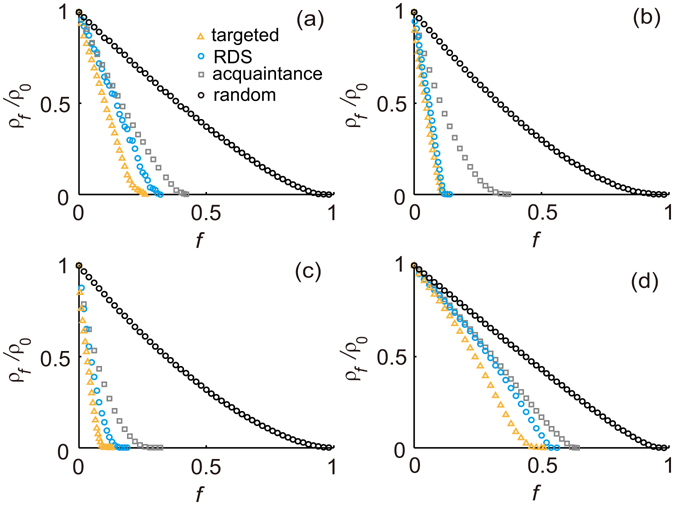
Reduced prevalence *ρ*_*f*_/*ρ*_0_ from simulations of the SIS model in (**a**) the Advogato network, (**b**) the Brightkite network, (**c**) the Epinions network and (**d**) the MSM network with random strategy, acquaintance strategy, targeted strategy and RDS strategy, at a fixed spreading rate *λ* = 0.25. The prevalence is averaged over 100 simulations.

To further verify the effectiveness of the RDS strategy, we implemented the strategy with four real-world social networks (Advogato network, Brightkite network, Epinions network and MSM network, see details in Materials and Methods). The averages of the cut-off degree *k*_*cut*_ obtained from simulations for each network are shown in Table 1. We can see that these estimated cut-off thresholds are very close to the values which is obtained from the real average degree and degree distribution of a network. The results of numerical simulations for the epidemic spreading on the four networks are shown in Fig. 3. The results are similar to those obtained for the BA network. For all networks, the *f*_*c*_ of RDS strategy is also closer to that of targeted strategy compared to acquaintance strategy and random strategy. Specifically, the difference in *f*_*c*_ for the RDS strategy as compared to the targeted strategy was only about 0.02 for the Advogato network, 0.01 for the Brightkite network, 0.05 for the Epinions network and 0.06 for the MSM network. These results well illustrate that the efficiency of RDS strategy is similar to that of the targeted strategy and much better than that of the acquaintance strategy and the random strategy in the real-world network.

**Table 1:** Statistics of experiment networks.

Network	*N*	*M*	<*k*>	*k*_cut_^* a^	${\overline{{\boldsymbol{k}}}}_{{\boldsymbol{cut}}}$ ^b^
BA network	10000	59980	5.998	11	11.230
Advogato network	5158	78852	15.287	9	8.952
Brightkite network	58109	427712	7.36	13	13.167
Epinions network	75877	811478	10.694	5	5.260
MSM network	16082	446170	27.743	14	13.700

^a^ *k*_cut_^*^ is obtained from the real average degree and degree distribution of a network.

^b^ ${\overline{k}}_{cut}$ is the average of 100 simulation results.

### RDS strategy with egocentric information

When the number of individuals we desire to immunize is large, the length of the RDS recruitment chain with the RDS strategy may be very long. Inspired by the conclusion that the RDS estimator integrated the egocentric information performs better than the others^47^, we can introduce such additional information into RDS strategy to shorten the length. The egocentric information is the data collected in respondents’ ego networks during RDS process, i.e, the property of the respondent’s contacts such as gender and the network size of the contacts. Specifically, when egocentric information about the network size of the respondents’ contacts is available, we can immunize the contact of the respondent if the contact has a network size over the threshold, i.e., ≥*k*_*cut*_.

**Figure 4:**
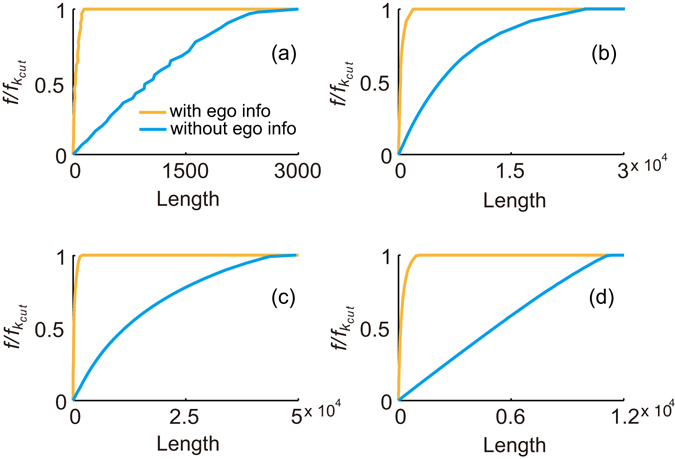
The length of the RDS chain from simulations that implemented the RDS strategy with and without egocentric information in (**a**) the Advogato network, (**b**) the Brightkite network, (**c**) the Epinions network and (**d**) the MSM network. *f*_*kcut*_ refers to the fraction of nodes whose degree *k* ≥ *k*_*cut*_. The length is averaged over 100 simulations.

As shown in Fig. 4, the length of RDS chain can be effectively shortened if egocentric information is collected. When immunizing all nodes whose degree *k* ≥ *k*_*cut*_, the length of RDS chain is shortened as 1/17 in Advogato network, 1/13 in Brightkite network, 1/28 in Epinions network, and 1/12 in MSM network. In real implementations, accurate egocentric information is difficult to collect because individuals may not know their neighbours’ degree well. However, this method is well suited for immunization on online social networks where the neighbours’ degree is easy to access, such as implementing online interventions based on social platforms, controlling and optimizing the dissemination of information or preventing the widely spread of rumour and incorrect information through the online networks.

### Estimation for the number of immunized nodes

In the real RDS recruitment process, the sample size of accessible samples is limited or hard to expand. We can only use the obtained samples to implement the RDS immunization strategy. Therefore, we concern about the RDS strategy’s efficiency of immunizing nodes from the samples, i.e., whether the number of immunized nodes reaches our desire goal by implementing our strategy within the existing sample with limited number of respondents.

Assuming that the RDS process is done with replacement (note that this assumption is based on the fact that sampling without replacement creates negligible bias compared with sampling with replacement in RDS when sample size is small^25, 26, 45^), the number of initial seed is 1 and the number of distributed coupon is 1, we approximate this immunization process with *n* Bernoulli trials. Then the probability of immunizing *w* nodes in *n* samples can be obtained by:

$$Pr(w)=(\begin{array}{c}n\\ w\end{array}){p}^{w}{(1-p)}^{n-w},$$

where *p* is the probability of immunizing nodes whose degree *k* ≥ *k*_*cut*_ in the population. Thus, the expectation of *w*:

1 $$E[w]=np\mathrm{.}$$

In RDS, the inclusion probability of a node *i* is proportional to its degree *k*_*i*_:

$${P}_{i}=\frac{{k}_{i}}{\sum _{j=1}^{N}{k}_{j}}.$$

Consequently, the inclusion probability of nodes whose degree *k* = *k*_*i*_ can be obtained by:

$$\begin{array}{l}{P}_{k={k}_{i}}={P}_{i}{n}_{{k}_{i}}=\frac{{k}_{i}}{\sum _{j=1}^{N}{k}_{j}}{n}_{{k}_{i}}=\frac{{k}_{i}}{ < k > N}\cdot {p}_{d}({k}_{i})N=\frac{{k}_{i}{p}_{d}({k}_{i})}{ < k > },\end{array}$$

where *n*_*ki*_ is the number of nodes whose degree *k* = *k*_*i*_ and *p*_*d*_(*k*_*i*_) is the population degree distribution. Hence the inclusion probability of nodes whose degree *k* ≥ *k*_*cut*_ can be obtained:

2 $$p=\frac{1}{ < k > }\sum _{k\ge {k}_{cut}}k{p}_{d}(k\mathrm{).}$$

**Figure 5:**
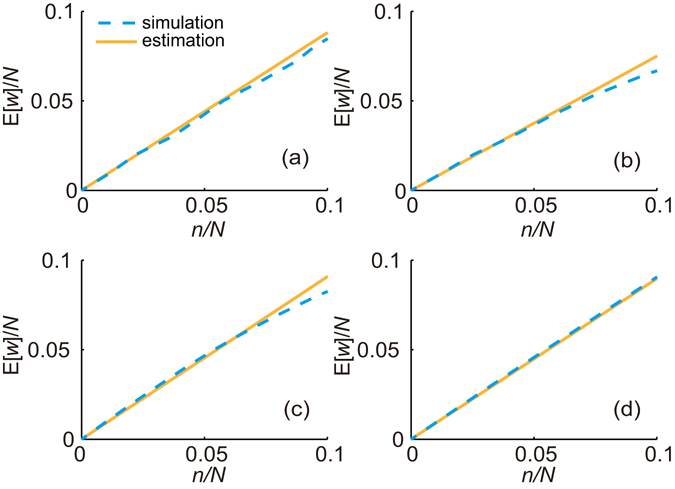
The expectation of the number of immunized nodes E[*w*] from the theoretical estimations and the immunization simulations in (**a**) the Advogato network, (**b**) the Brightkite network, (**c**) the Epinions network and (**d**) the MSM network. The expectation is averaged over 100 simulations.

Finally, substituting equation (2) into equation (1), we can obtain the theoretical estimation of E[*w*] at a given *n*. To validate this conclusion, we compare the theoretical estimations and immunization simulations in the four real-world networks. Figure 5 shows that the theoretical estimations of E[*w*] are almost consistent with the simulation results when the sample size is smaller than 10 percent of the whole population. The deviations between them increase gradually with the sample size due to the effect of sampling without replacement.

### A practical application of RDS strategy

**Figure 6:**
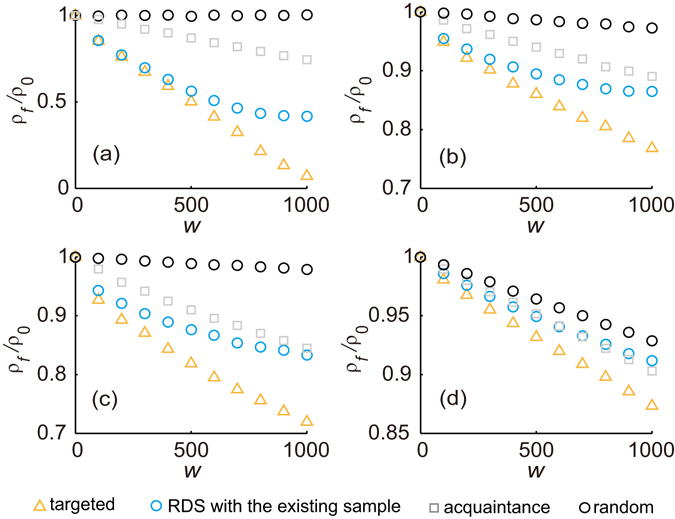
Reduced prevalence *ρ*_*f*_/*ρ*_0_ from simulations of the SIS model in (**a**) the Advogato network, (**b**) the Brightkite network, (**c**) the Epinions network network and (**d**) the MSM network with random strategy, acquaintance strategy, targeted strategy and practical RDS strategy, i.e., immunization within the existing sample, at a fixed spreading rate *λ* = 0.25. The prevalence is averaged over 100 simulations.

In simulations of the model network and real-world social networks we implement the RDS strategy to immunize large enough number of individuals from sufficient samples for the eradication of epidemics. This is performed to compare the efficiency of different strategies. However, in the real implementation on hidden populations, it may be difficult to immunize the desired number of eligible individuals from the RDS samples because sample size may be limited or small, and the sample may be difficult to expand. Therefore, we can adopt a more practical approach: Immunize within the existing sample (such as 1000 RDS participants) according to the participants’ ranking order of their reported degree in the sample. Under such setting, the results of numerical simulations for epidemic spreading among the four networks are shown in Fig. 6. We can see that the reduced prevalence *ρ*_*f*_/*ρ*_0_ of such a practical RDS strategy performs similar to acquaintance strategy at different values of *w* (the number of immunized nodes) in the MSM network and better than acquaintance strategy in other three networks. Although the reduction of the prevalence with this practical RDS strategy is much lower than with the targeted strategy when immunizing the same number of nodes, it is clear that the traditional strategies are less applicable for hidden populations than the proposed strategy which is combined with the RDS sampling process.

## Discussion and Conclusion

In this study, we propose an immunization strategy based on the RDS process, which is a sampling methodology currently widely adopted and applied in the study of hidden populations worldwide. The proposed RDS strategy provides a systematical approach and overcomes difficulties for hidden population intervention and immunization. It can be implemented along with existing RDS studies and so that makes accessing to hidden populations possible during the immunization process. furthermore, the simulation results indicate that its efficiency is just following that obtained with the targeted strategy which requires comprehensive global information for the population, and much better than that obtained with the acquaintance strategy and random strategy for the eradication of epidemics.

The advantage of RDS immunization strategy for hidden populations is obvious: First, existing methodologies has been developed to estimate average degree and degree distribution from an RDS sample. In the study of hidden populations, such estimates provide crucial inferences related to global information and can be used to obtain the theoretical immunization threshold. Second, the immunization can be well combined with the existing RDS sampling process. After the sample is obtained, individuals to be intervened or immunized are chosen among the sampled respondents. *Such an immunization process based on RDS avoids randomly selecting initial individuals, which is the first step for most traditional local strategies but impractical for hidden populations*. Meanwhile, RDS process can select key individuals with high connections more quickly than the random selection due to the fact that the inclusion probability of an individual in RDS is proportion to its degree^25, 26^; On this basis, with the use of the cut-off threshold *k*_*cut*_ which is obtained from the immunization threshold of targeted immunization, the higher degree individuals can be immunized almost as quickly as the targeted immunization (see Supplementary Fig. S1 online). That’s also the reason why the efficiency of RDS strategy is closely following that obtained with targeted strategy, and better than that obtained with the acquaintance strategy and random strategy in the simulations. Third, the advantages of RDS make immunization process more feasible and cost-effectiveness. (1) The incentive mechanism of RDS improves recruiting efficiency. Each pair of individuals successfully recruited in RDS will be both rewarded. The rewards can stimulate respondents to pass their coupons on and encourage peer participation. (2) The peer-driven design allows individuals received the coupon to decide for themselves whether to participate. This makes recruited respondents more likely to cooperate. (3) RDS implementation is an automatic process. After selecting the seed, the recruitment process will continue automatically till the desired sample size reaches. The researchers only need to interview the respondents at fixed locations.

For the proposed strategy, the efficiency of the immunization, i.e., the critical immunized fraction *f*_*c*_ is largely determined by *k*_*cut*_. To guarantee the credibility of the obtained *k*_*cut*_, reliable estimations of average degree and degree distribution are needed. The estimations used in this paper are considered reliable^38^ and the estimation results on BA network confirm this conclusion as well. When obtaining the value of *k*_*cut*_ for the eradication of epidemics, we assume that the spreading rate *λ* is known when medical experts have the means to obtain *λ* before the development of vaccines and the implementation of immunization. When *λ* is difficult to obtain, we can immunize those top-ranking nodes if possible. For example, our goal is to immunize the top 10% of nodes as more as possible. In this case, we can also calculate the value of *k*_*cut*_ by the percentage of top-ranking nodes we want to immunize (see Materials and Methods).

To conclude, the proposed RDS strategy shows great advantages on the immunization of hidden populations. First, it can be combined with the RDS sampling process (i.e., no extra sample selection is needed for immunization), which makes immunization and intervention on hidden populations possible and effective. Second, the selection criteria for individuals to be immunized is based on comparison of respondents’ degree with the estimated cut-off degree *k*_*cut*_, i.e., calculation for global ranking of individuals are not required. Third, with only local information, the RDS strategy has efficiency similar to that of the targeted strategy. We believe that the proposed method offers a practical strategy for designing and improving of hidden population intervention programmes, in conjunction with current RDS sampling studies, the efficacy and cost-effectiveness of immunization could be improved significantly.

## Materials and Methods

### Networks

The underlying networks for the study of infectious disease transmission have rarely been the actual physical network, due to difficulties in obtaining such data. Instead, the model networks and social contact networks are most often used^48, 49^. And it is still an open question how well the example networks used represent the structure of real contact networks, e.g., the efforts to model contact networks of IDUs^50^.

In this paper, We test the proposed RDS strategy on a Barabási-Albert model and four real social contact networks. The BA network is generated by the algorithm devised in ref. 46: The number of starting nodes *m*_0_ is 5 and the number of new links *m* at every time step is 3. The four real-world networks were the Advogato online social network^51^, the Brightkite online social network^52^, the Epinions who-trust-whom online social network^53^ and the anonymized online social MSM network^41, 45, 54^. When implementing the RDS, it is assumed that the social network of the population is undirected. Therefore, we regard all edges of the networks as undirected. In order to make sure each node could be recruited with simulated RDS, we obtain four experiment networks by keeping members of the giant connected component (GCC) from above four undirected networks. The basic statistics of these experiment networks are shown in Table 1.

### Estimate of degree distribution and average degree

In implementing the RDS process, we collected the degree of each respondent in our sample. According to the sample degree distribution *q*_*d*_(*k*), the population degree distribution *p*_*d*_(*k*) can be estimated as^38^

3 $${\widehat{p}}_{d}(k)=\frac{\frac{1}{k}\cdot {q}_{d}(k)}{\sum _{k=1}^{\max (k)}\frac{1}{k}\cdot {q}_{d}(k)}.$$

Then the average degree <*k*> can be estimated as^38^

4 $$ < \widehat{k} > =\sum _{k=1}^{\max (k)}k\cdot {\widehat{p}}_{d}(k\mathrm{).}$$

### Approximate solution for immunization threshold

The approximate solution for the immunization threshold of scale-free networks with an arbitrary connectivity exponent in the case of targeted immunization is ref. 4

5 $${g}_{c}\simeq \exp (-2/m\lambda ),$$

where *g*_*c*_ is the immunization threshold, *m* = <*k*>/2 and *λ* is the spreading rate in the network.

### Obtain the value of *k*_*cut*_

(1) Obtain *k*_*cut*_ for the eradication of the epidemics. If we want that the strategy works approximately well to targeted immunization for the eradication of epidemics, the top $\widehat{{g}_{c}}\cdot N$ nodes should be immunized ($\widehat{{g}_{c}}$ is the estimation from equation (5). In the cumulative degree distribution *p*_*c*_(*k*), we can find a cut-off degree ${k}_{cut}=\,{\rm{\max }}\,\{k|{p}_{c}(k)\le 1-\widehat{{g}_{c}}\}$. In this case, we assume that the spreading rate *λ* is known. (2) Obtain *k*_*cut*_ for immunizing top *a*% of nodes. In this case, the value of *λ* is not needed. We can directly find a cutoff degree ${k}_{cut}=\,{\rm{\max }}\,\{k|{p}_{c}(k)\le 1-a \% \}$ in the estimated cumulative degree distribution.

### Immunization strategies used for comparison

#### Random immunization

The random immunization, or uniform immunization, is a very simple immunization procedure by randomly selection of individuals in a population. This strategy needs to immunize a very large fraction of individuals in the scale-free network in order to eradicate the epidemics.

#### Targeted immunization

The targeted immunization is a most efficient strategy based on the ranking order of the individuals’ number of contacts (degree). When the global information, i.e., the degree of each individual, is available, this degree ranking order is easily obtained and the most connected individuals are immunized in turn from the ranking order. Although the targeted immunization can target the influential spreaders, i.e., the most connected individuals, the global information are hard to gather for the general population, not to mention hidden populations which have strong privacy concerns.

#### Acquaintance immunization

In the acquaintance immunization, a certain number of individuals are randomly selected and then a random acquaintance of each of these individuals is selected to immunize. This strategy only need to know the random chosen individual and the acquaintances in his or her contact so that it overcomes the requirement of global information. The acquaintance immunization is impractical for hidden populations because the first step of this strategy, i.e., obtaining random samples, cannot be implemented on the population which is lack of the sampling frame.

#### RDS strategy

In this paper, we proposed a systematically approach for hidden population immunization called RDS strategy. Specifically, this strategy is consisted of the following steps. First, using the collected degree of each respondent recruited through RDS to estimate the degree distribution and average degree of the studied population by equation (3) and equation (4). According to the estimated average degree, the approximate immunization threshold $\widehat{{g}_{c}}$ in case of targeted immunization can be obtained by equation (5). Then in the cumulative degree distribution *p*_*c*_(*k*) obtained from the estimated degree distribution, we can find a ‘cut-off degree’ ${k}_{cut}=\,{\rm{\max }}\,\{k|{p}_{c}(k)\le 1-\widehat{{g}_{c}}\}$. Lastly, the RDS participants whose degree *k* ≥ *k*_*cut*_ are immunized.

The proposed RDS strategy can be combined with the RDS sampling process and doesn’t require the random selection of initial individuals as part of the first step which is needed for the acquaintance immunization. Its selection criteria for individuals to be immunized is based on comparison of respondents’ degree with the estimated cut-off degree so that it doesn’t need the calculation for global ranking of individuals which is required for the targeted immunization.

### Simulations of epidemic spread

Several models have been proposed for studying the behaviours of epidemic dynamics in networks. In this paper, we focus on the standard Susceptible-Infected-Susceptible model^55^. In the SIS model, each node of the network represents an individual, and each edge is a connection through which the infection can spread. A node can be in one of the two states: susceptible or infected. In simulations of this paper, the SIS spreading processes are implemented by using synchronous updating methods. Namely, in each time step *t*, each susceptible node is infected with probability *v* if it is connected to one or more infected nodes. At the same time, all infected nodes are cured and become susceptible with probability *δ*. Time increases by Δ*t* = 1, and the dynamical process terminates when the system reaches a steady state. The spreading rate for the epidemic is then defined as *λ* = *v*/*δ*.

## Electronic supplementary material

Supplemental Information
