# Supplementary material for: An Immunization Strategy for Hidden Populations
Source: Sci Rep. 2017 Jun 12;7:3268. doi: 10.1038/s41598-017-03379-4 (PMC5468297; doi:10.1038/s41598-017-03379-4)
Supplement: Supplementary file 1 — Supplemental Information [file 41598_2017_3379_MOESM1_ESM.pdf]

# *Supplemental Information:* An Immunization Strategy for Hidden Populations

Saran Chen[1] and Xin Lu[1,2,3,4*]

[1]College of Information System and Management, National University of Defense Technology, 410073 Changsha, P.R. China

[2]School of Mathematics and Big Data, Foshan University, 528000 Foshan, P.R.China

[3]Department of Public Health Sciences, Karolinska Institutet, 17177 Stockholm, Sweden

[4]Division of Infectious Disease, Key Laboratory of Surveillance and Early Warning on Infectious Disease, Chinese Centre for Disease Control and Prevention, Beijing 102206, P.R. China

[*]xin.lu@flowminder.org

# Ⅰ The speed of immunizing top nodes

We calculated the top ranked nodes as a fraction of nodes considered in each immunization strategy. Fig. S1 shows that the RDS strategy can immunize the high degree individuals (the top 10% individuals in the ranking of degree) almost as quickly as targeted immunization, and is much more quickly than acquaintance immunization and random immunization.

In RDS, the inclusion probability of a node is proportional to its degree in RDS; therefore high degree nodes can be sampled more quickly in RDS than in the random selection; Meanwhile, the cut-off threshold which is obtained based on the immunization threshold of targeted immunization can partition the sampled nodes into two parts; in this way, the nodes with higher degree in the sample can be targeted almost as quickly as targeted immunization.

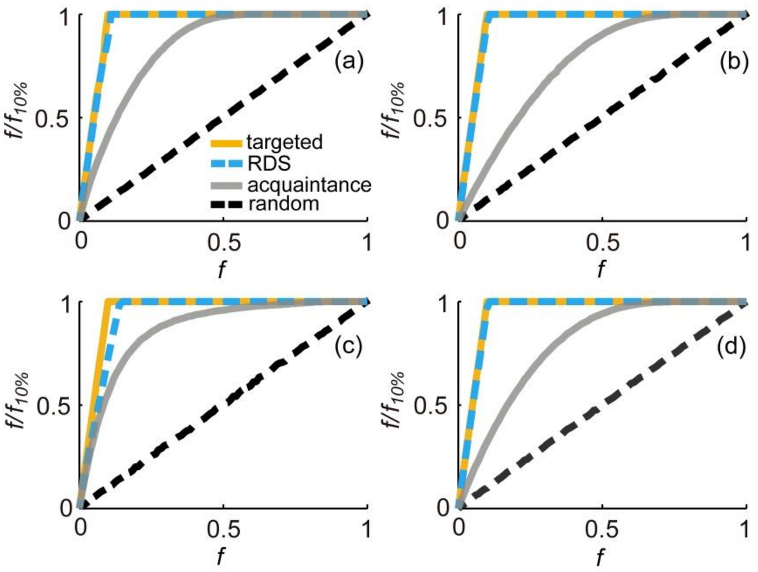


**Fig. S1** The speed of immunizing top 10% individuals in (a) the Advogato network , (b) the Brightkite network , (c) the Epinions network and (d) the MSM network. *f* refers to the fraction of immunized nodes and $f_{10\%}$ refers to the fraction of nodes whose degree in the top 10%. The results are averaged over 100 simulations.

# Ⅱ immunization in Watts-Strogatz (WS) network and Erdős–Rényi (ER) network

In the main article, we implemented the RDS strategy in the Barabasi-Albert (BA) network due to the fact that the most social networks in real world are scale-free networks, i.e., heterogeneous networks. And compared to the homogeneous network

in which each node has approximately the same degree, the heterogeneous network is prone to the spreading and the persistence of infections because of its diverging connectivity fluctuations[1][2].

Beside the Barabasi-Albert (BA) network used in the main article, we have implemented the RDS strategy in another two classical network models, i.e., the Watts-Strogatz (WS) network and Erdős–Rényi (ER) network. The WS network is constructed in consistence with the study in [3]: The starting point is a ring with 10000 nodes, in which each node is symmetrically connected with its 10 nearest neighbors; Then, for every node each edge connected to a clockwise neighbor is kept as originating from the original node and rewired to a randomly chosen target node with probability 0.5. The ER network is generated by the algorithm in [4]: it starts with 10000 nodes and each pair of nodes is connected randomly with probability 0.001.

Fig. S2 shows the simulation results that the reduced prevalence $\rho_f / \rho_0$ varies with the number of the immunized nodes. The efficiency of RDS strategy is also following that obtained with the targeted strategy and better than that obtained with the acquaintance strategy and random strategy. However, the difference of these efficiencies is not very remarkable; we can see the immunization thresholds of the four immunization strategies are almost equivalent. The results are consistent with the conclusion in Pastor's research [3] which is verified that in case of immunizing on the homogeneous networks (e.g., the Watts-Strogatz (WS) network) the immunization threshold is almost equivalent in the random and targeted immunization.

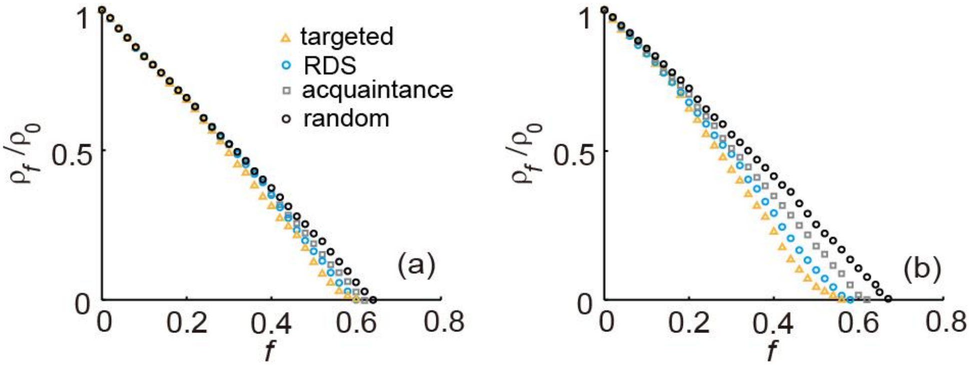


**Fig. S2** Reduced prevalence $\rho_f / \rho_0$ from simulations of SIS model in WS network (a) and ER network (b) at a fixed spreading rate λ=0.25. The RDS strategy is implemented with 1 seed and 1 coupon. The cut-off threshold $k_{cut}$ is used the estimated average degree obtained from samples. The prevalence is averaged over 100 simulations.

## Ⅲ Immunization with multiple seeds and coupons

Besides implementing RDS strategy with 1 seed and 1 coupon in main article, we have explored the effect of increasing seeds and coupons. Fig. S3 and Fig. S4 show that the efficiency of the RDS strategy varies from the different number of seeds and coupons. We can see that the efficiency of RDS strategy is not affected by the seed number or coupon number. That's because the number of seeds or coupons does not change the inclusion probability of the individuals[5-7].

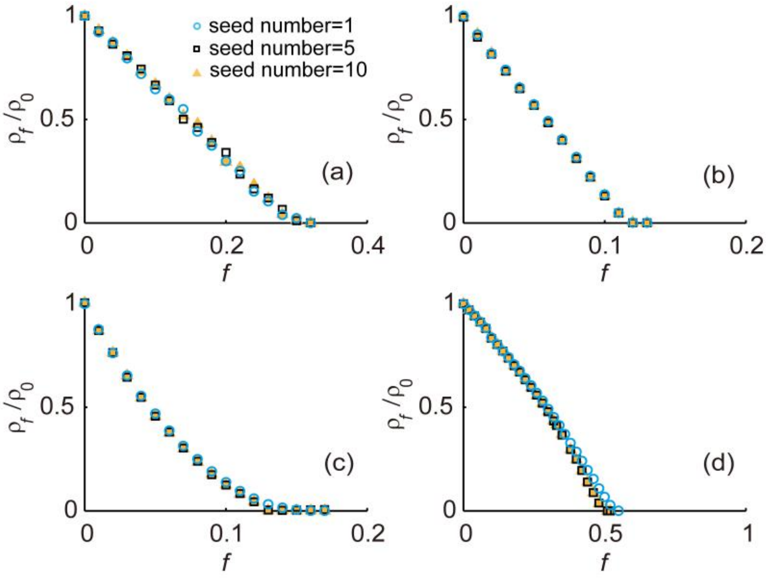


**Fig. S3** Reduced prevalence $\rho_f/\rho_0$ from simulations of SIS model in (a) the Advogato network, (b) the Brightkite network, (c) the Epinions network and (d) the MSM network at a fixed spreading rate $\lambda$=0.25. The number of coupon is 1. The prevalence is averaged over 100 simulations.

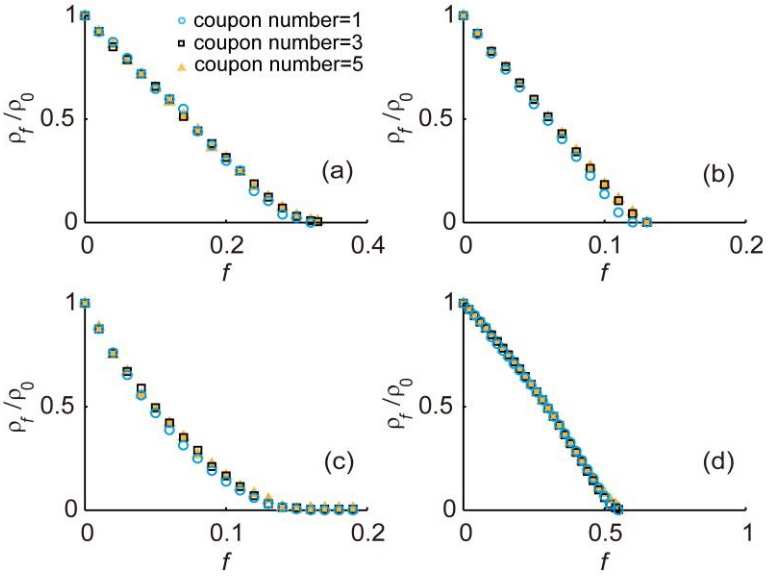


**Fig. S4** Reduced prevalence $\rho_f/\rho_0$ from simulations of SIS model in (a) the Advogato network , (b) the Brightkite network , (c)the Epinions network and (d) the MSM network at a fixed spreading rate $\lambda$=0.25. The number of seeds is 1. The prevalence is averaged over 100 simulations.

## Ⅳ General method for reducing the length of the referral chain

The general method for reducing the length of the referral chain is increasing the number of seeds or coupons. The results of implementing RDS with different number of seeds and coupons are shown in Fig. S5. We can see that the two general methods can reduce the length of the referral effectively. However, in practice we usually have only a small number of seeds in the implementation due to the fact that the seeds typically are the current or former members of the targeted population (e.g., the current IDUs). Meanwhile, the rejection rate of distributing coupon is usually high in practice, which makes long referral chains rarely appear.

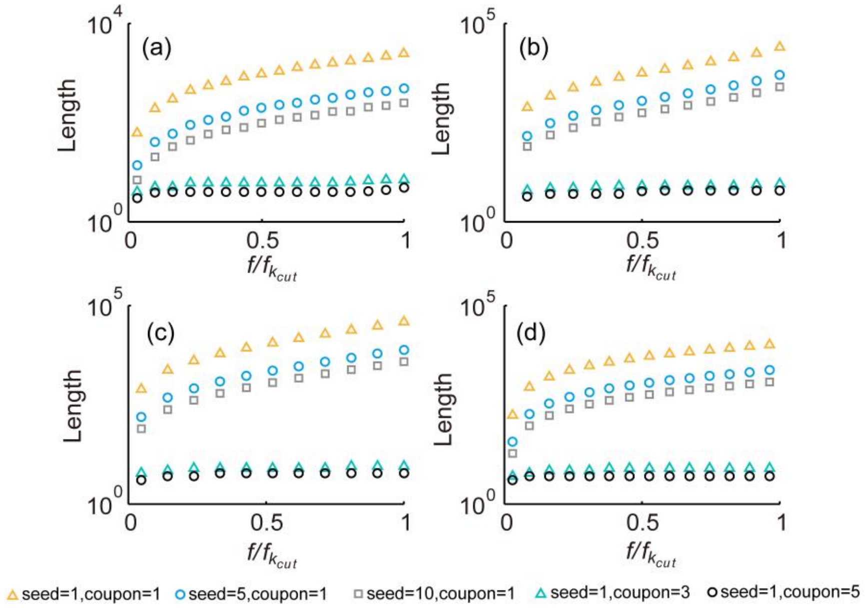


**Fig. S5** The length of the RDS chain from simulations that implemented with different number of seeds and coupons in the Advogato network (a), the Brightkite network (b), the Epinions network (c) and the MSM network (d) at a fixed spreading rate λ=0.25. The results are averaged over 100 simulations.

## References

[1] Pastor-Satorras, R., Vázquez, A. & Vespignani, A. Dynamical and correlation properties of the internet. *Physical Review Letters* **87**, 527–537 (2001).
[2] Pastor-Satorras, B. R. Vespignani a: Epidemic dynamics and endemic states in complex networks. In *Phys Rev E Stat Nonlin Soft Matter Phys 2001, E63(Pt 2):066117* (2010).
[3] Pastor-Satorras, R. & Vespignani, A. Immunization of complex networks. Physical Review E Statistical Nonlinear & Soft Matter Physics 65, 106–126 (2002).
[4] Erdős, P, Rényi, A. On the evolution of random graphs[J]. Publication of the Mathematical Institute of the Hungarian Academy Ofences, 1960, 38(1):17--61.
[5] Heckathorn, D. D. Respondent-driven sampling: A new approach to the study of hidden populations. Australasian Radiology 40, 206–212 (1997).
[6] Heckathorn, D. D. Respondent-driven sampling ii: Deriving valid population estimates from chain-referral samples of hidden populations. *Social Problems* **49**, 11–34 (2002).
[7] Volz, E. & Heckathorn, D. D. Probability based estimation theory for respondent driven sampling. *Journal of Official Statistics* **24**, 79–97 (2008).
